# Supplementary material for: PQS and pyochelin in Pseudomonas aeruginosa share inner membrane transporters to mediate iron uptake
Source: Microbiol Spectr. 2024 Jan 3;12(2):e03256-23. doi: 10.1128/spectrum.03256-23 (PMC10846271; doi:10.1128/spectrum.03256-23)
Supplement: Supplemental material — Fig. S1 to S7 and Tables S1 to S3. [file spectrum.03256-23-s0001.pdf]

Figure S1

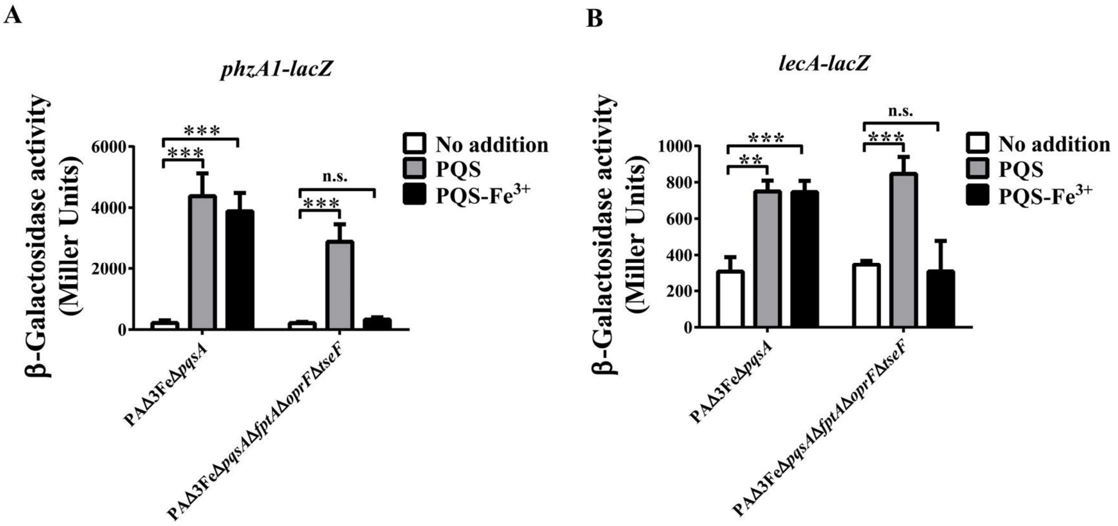

Fig S1 The effect of exogenous addition of PQS and PQS-Fe^{3+}on the expression of *phzA1* and *lecA*. Cells were grown in TSB broth with or without 40 μM PQS or PQS-Fe^{3+}(PQS: Fe^{3+}=3:1). Levels of *phzA1* and *lecA* transcription in *P. aeruginosa* mutant strains PAΔ3FeΔ*pqsA* and PAΔ3FeΔ*pqsA*Δ*fptA*Δ*oprF*Δ*tseF* were monitored using the *phzA1–lacZ* and *lecA*–lacZ transcriptional fusions, respectively. The graphs show the mean and standard deviation of three experiments performed in five replicates each time. **, *P*<0.01; ***, *P*<0.001; n.s., not significant.

Figure S2

A

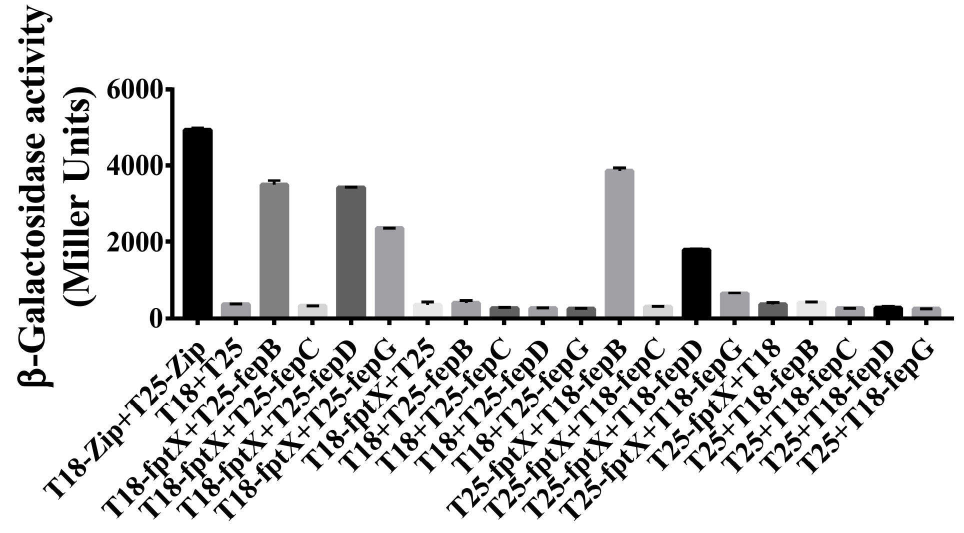

B

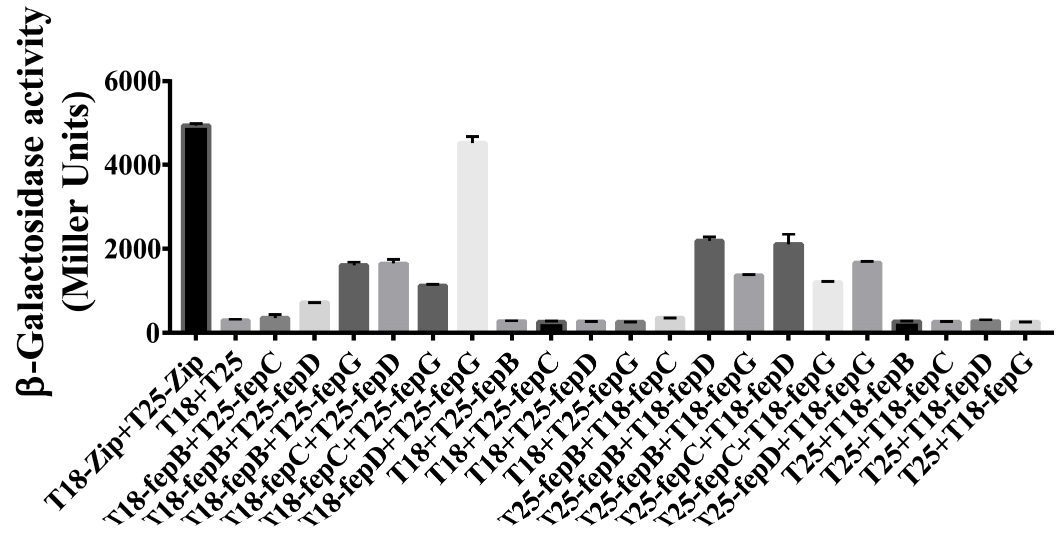

Fig S2 Interactions between FptX, FepB, FepC, FepD and FepG identified by bacterial two-hybrid experiments. The β-galactosidase activity of co-transformants on MacConkey agar plates were measured. (A): Relevant to Fig. 1D, (B): Relevant to Fig. 1E. The plasmid combination is displayed below the graphs. The graph shows the mean and standard deviation of three experiments performed in five replicates each time.

Figure S3

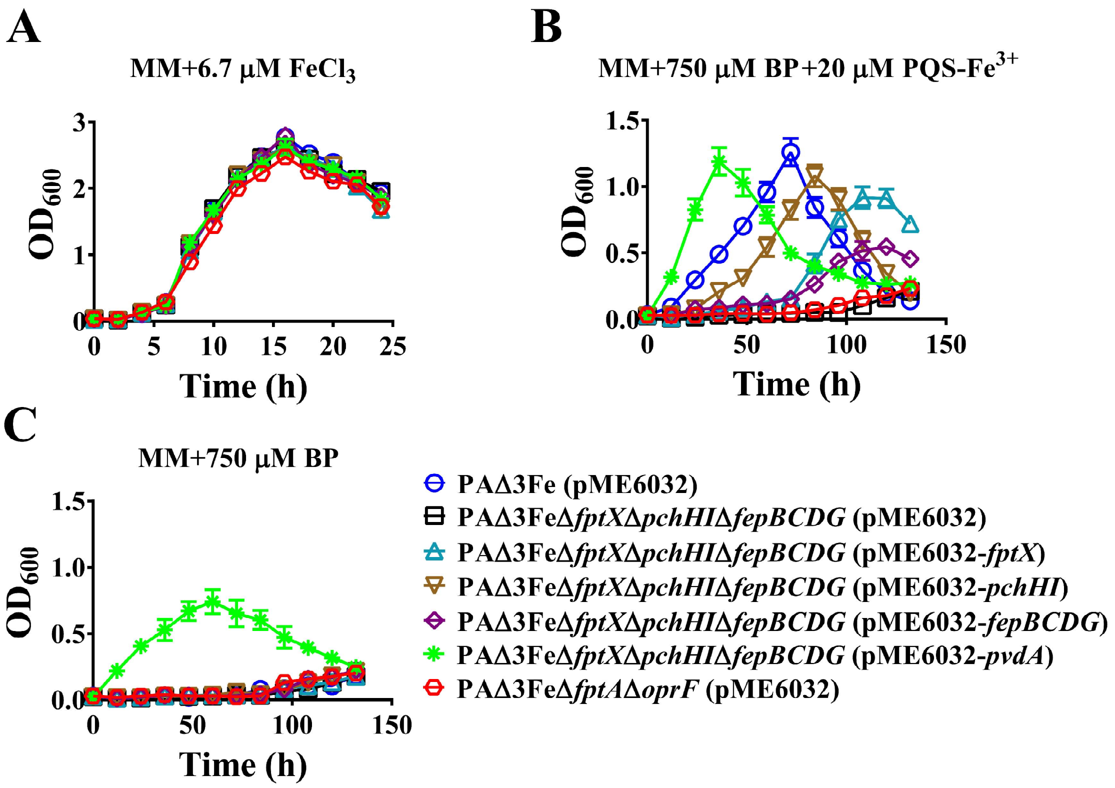

Fig S3 Effects of complementary *fptX*, *pchHI* and *fepBCDG* on the growth of *P. aeruginosa* mutant PAΔ3FeΔ*fptX*Δ*pchHI*Δ*fepBCDG*, respectively. Conditions of experiment were similar to Fig. 1F-H. Complementary *pvdA* was used as a positive control. All the data represent the results of at least three independent experiments. The error bars represent the standard deviations.

Figure S4

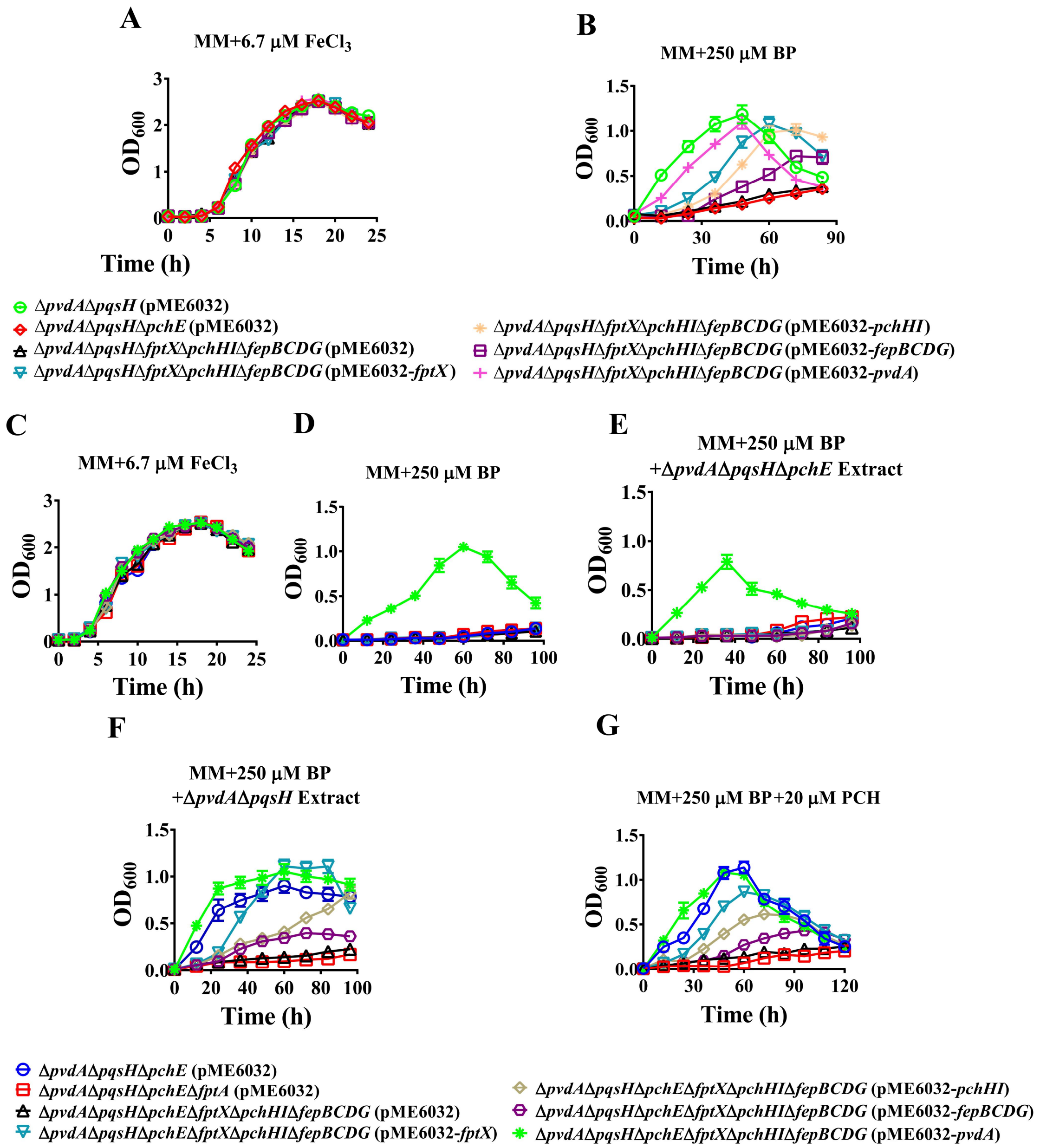

Fig S4 Effects of complementary *fptX*, *pchHI* and *fepBCDG* on the growth of *P. aeruginosa* mutants Δ*pvdA*Δ*pqsH*Δ*fptX*Δ*pchHI*Δ*fepBCDG* and Δ*pvdA*Δ*pqsH*Δ*pchE*Δ*fptX*Δ*pchHI*Δ*fepBCDG*, respectively. Conditions of experiment were similar to Fig. 2. Complementary *pvdA* was used as a positive control. All the data represent the results of at least three independent experiments. The error bars represent the standard deviations.

Figure S5

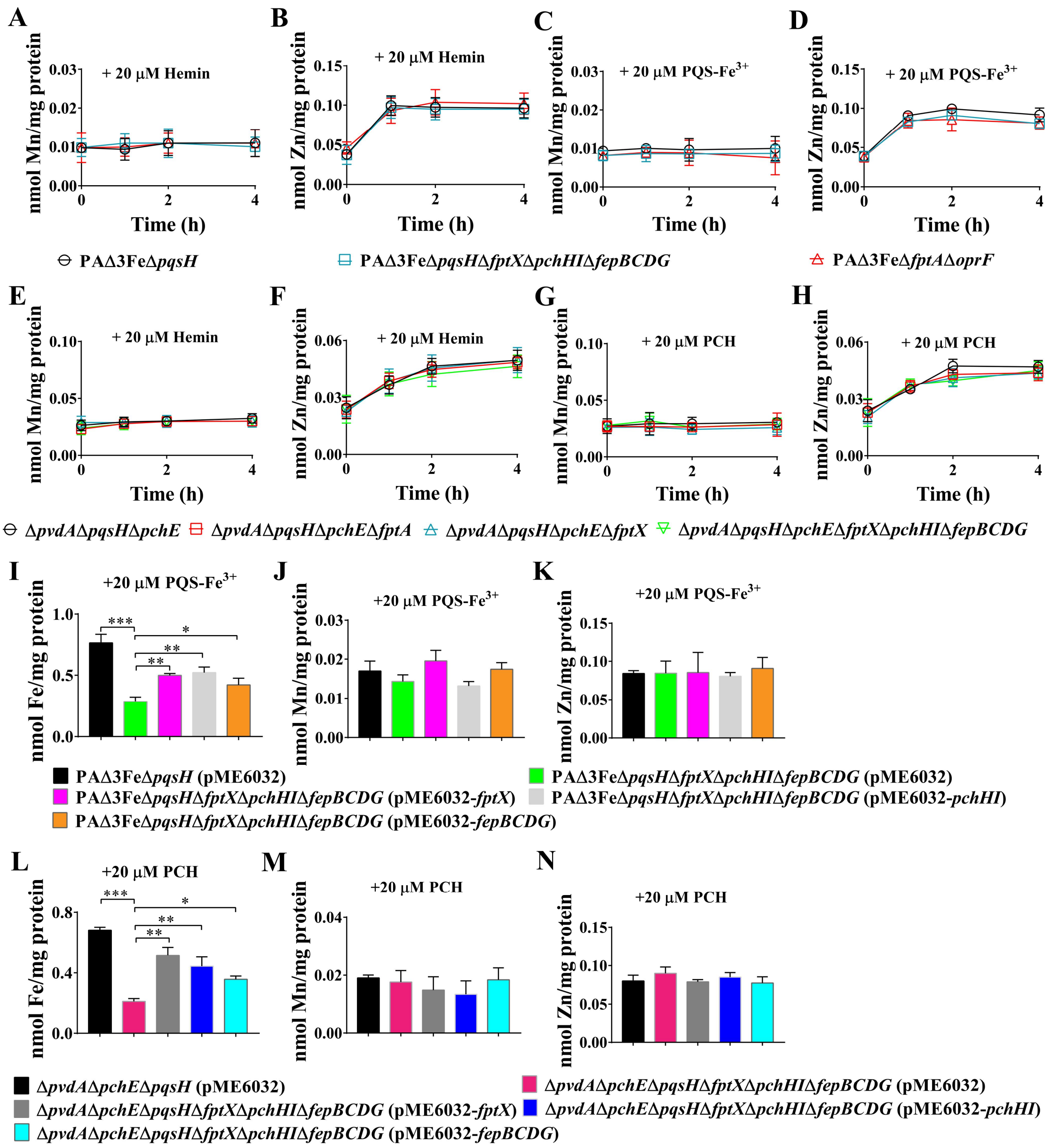

Fig S5 Monitoring of intracellular metal content in *P. aeruginosa*. (A-D) Relevant to Fig. 3E-F. (A-D) *P. aeruginosa* mutants PAΔ3FeΔ*pqsH* and PAΔ3FeΔ*pqsH*Δ*fptX*Δ*pchHI*Δ*fepBCDG* were cultured in the MM to mid-log phase, and the cells were collected and suspended in phosphate buffered saline (PBS), then added 0.4% glucose and 20 μM hemin or PQS-Fe^{3+} (PQS:Fe^{3+}=3:1), and incubated at 37°C, 200 rpm for 0 h, 1 h, 2 h, and 4 h, respectively. The cell samples were collected and the intracellular metal ion content was determined by Inductively Coupled Plasma Mass Spectrometry (ICP-MS). (A, B): Adding 20 μM hemin in PBS, (C, D): Adding 20 μM PQS-Fe^{3+} (PQS:Fe^{3+}=3:1) in PBS, (A, C): Measurement of manganese ion content, (B, D): Measurement of zinc ion content. PAΔ3FeΔ*fptA*Δ*oprF* strain serves as the negative control. (E-H) Relevant to Fig. 3G-H. (E-H): *P. aeruginosa* mutants Δ*pvdA*Δ*pqsH*Δ*pchE*, Δ*pvdA*Δ*pqsH*Δ*pchE*Δ*fptX* and Δ*pvdA*Δ*pqsH*Δ*pchE*Δ*fptX*Δ*pchHI*Δ*fepBCDG* were cultured in the MM to mid-log phase, and the cells were collected and suspended in PBS, then added 0.4%

glucose and 20 μM hemin or pyochelin, and incubated at 37°C, 200 rpm for 0 h, 1 h, 2 h, and 4 h, respectively. The cell samples were collected and the intracellular metal ion content was determined by ICP-MS. (E, F): Adding 20 μM hemin in PBS, (G, H): Adding 20 μM PCH in PBS, (E, G): Measurement of manganese ion content, (F, H): Measurement of zinc ion content. Δ*pvdA*Δ*pqsH*Δ*pchE*Δ*fptA* strain serves as the negative control. (I-K): *P. aeruginosa* PAΔ3FeΔ*pqsH*Δ*fptX*Δ*pchHI*Δ*fepBCDG* strain and its corresponding complementary strains were cultured in the MM to mid-log phase, and the cells were collected and suspended in PBS, then added 0.4% glucose and 20 μM PQS-Fe^{3+} (PQS:Fe^{3+}=3:1), and incubated at 37°C, 200 rpm for 4 h, respectively. The cell samples were collected and the intracellular metal ion content was determined by ICP-MS. (I): Measurement of iron ion content, (J): Measurement of manganese ion content, (K): Measurement of zinc ion content. (L-N): *P. aeruginosa* Δ*pvdA*Δ*pqsH*Δ*pchE*Δ*fptX*Δ*pchHI*Δ*fepBCDG* strain and its corresponding complementary strains were cultured in the MM to mid-log phase, and the cells were collected and suspended in PBS, then added 0.4% glucose and 20 μM pyochelin, and incubated at 37°C, 200 rpm for 4 h, respectively. The cell samples were collected and the intracellular metal ion content was determined by ICP-MS. (L): Measurement of iron ion content, (M): Measurement of manganese ion content, (N): Measurement of zinc ion content. All data represent the results of at least three independent experiments. Error bars represent standard deviations. *, $P<0.05$, **, $P<0.01$, ***, $P<0.001$.

Figure S6

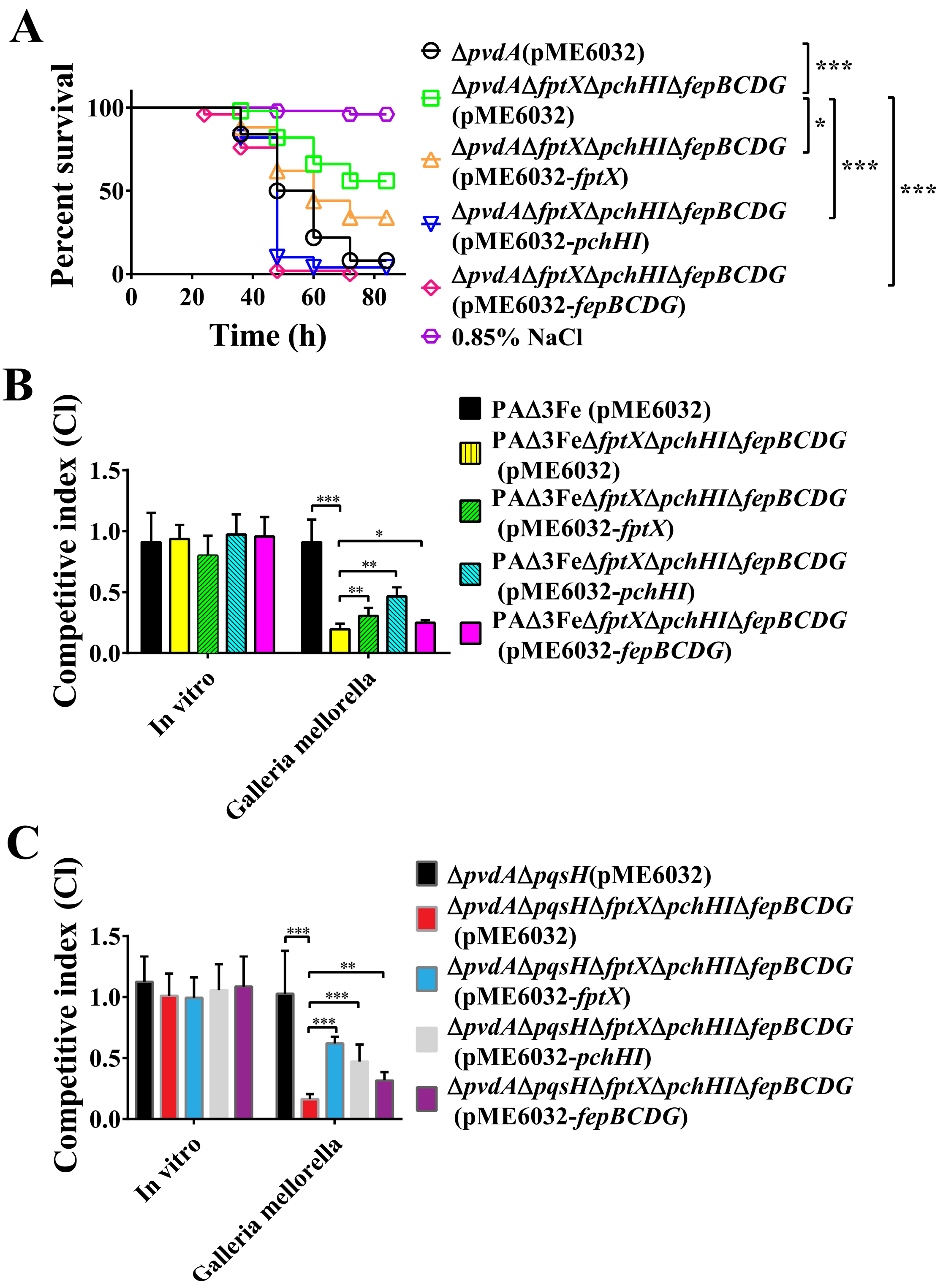

Fig S6 Effects of complementary *fptX*, *pchHI* and *fepBCDG* on the virulence of *P. aeruginosa* mutants Δ*pvdA*Δ*fptX*Δ*pchHI*Δ*fepBCDG*, PAΔ3FeΔ*fptX*Δ*pchHI*Δ*fepBCDG* andΔ*pvdA*Δ*pqsH*Δ*fptX*Δ*pchHI*Δ*fepBCDG*, respectively. Conditions of experiment were similar to Fig. 6. All data represent the results of at least three independent experiments. Error bars represent

standard deviations. *, $P<0.05$, **, $P<0.01$, ***, $P<0.001$.

Figure S7

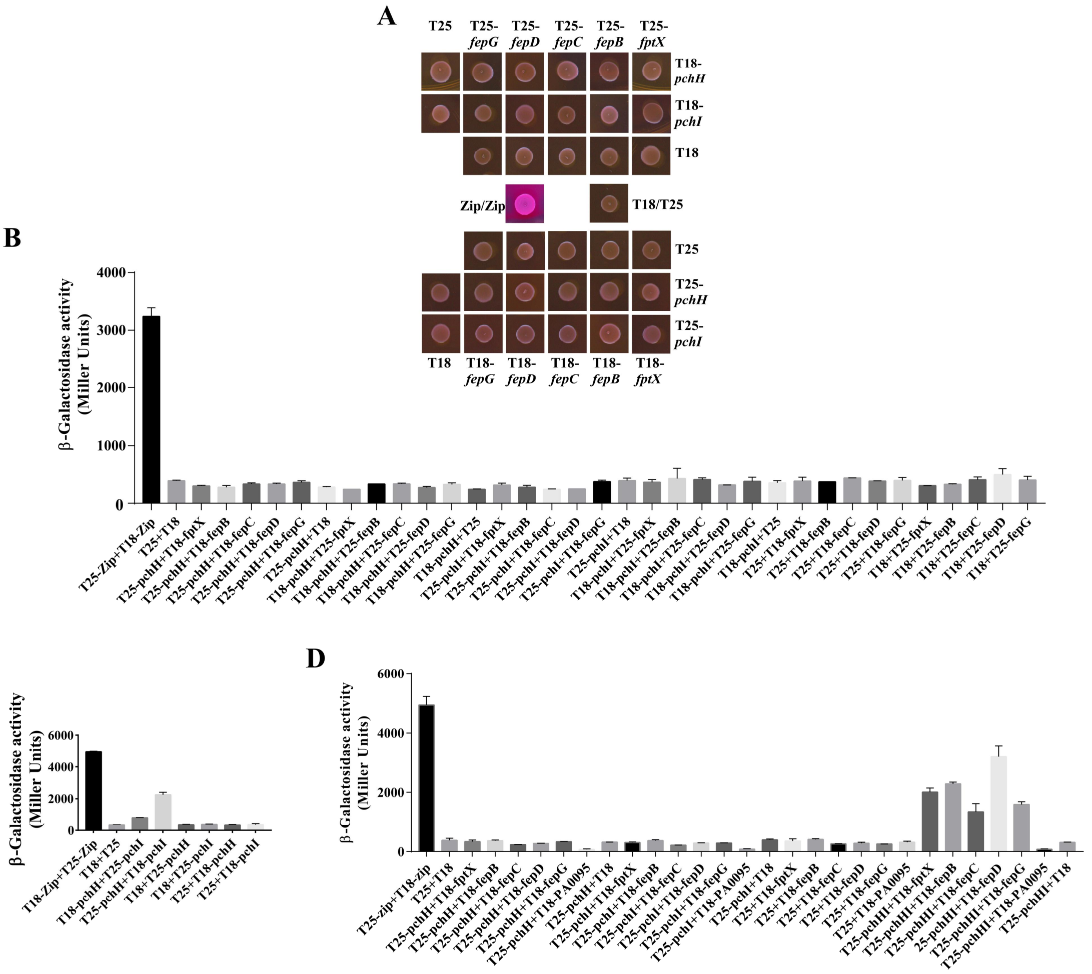

Fig S7 Interactions between FptX, PchHI and FepBCDG identified by bacterial two-hybrid experiments. Conditions of experiment were similar to Fig. 7. (A): There is no interaction between PchH and PchI with FptX, FepB, FepC, FepD and FepG, respectively. (B, C, D): The β-galactosidase activity of co-transformants on MacConkey agar plates were measured. (B) Relevant to A, (C)Relevant to Fig. 7A, (D)Relevant to Fig. 7B. The plasmid combination is displayed below the graphs. All data represent the results of at least three independent experiments. Error bars represent standard deviations.

Table S1 List of strains and plasmids used in this study

Strains	Characteristics	Source
Pseudomonas aeruginosa		
PAO1 (ATCC15692)	Wild-type	(1)
Δ*fptA*	*fptA* deletion mutant in PAO1	This study
Δ*fptX*	*fptX* deletion mutant in PAO1	This study
Δ*fptX*Δ*fepBCDG*	*fptX*/*fepBCDG* deletion mutant in PAO1	This study
Δ*fptX*Δ*pchHI*	*fptX*/*pchHI* deletion mutant in PAO1	This study
Δ*fptX*Δ*pchHI*Δ*fepBCDG*	*fptX*/*pchHI*/*fepBCDG* deletion mutant in PAO1	This study
Δ*fptX*Δ*pchHI*Δ*fepBCDG*Δ*pchR*	*fptX*/*pchHI*/*fepBCDG*Δ*pchR* deletion mutant in PAO1	This study
Δ*pvdA*Δ*pchE*Δ*feoB* (PAΔ3Fe)	*pvdA*/*pchE*/*feoB* triple deletion mutant in PAO1	(1)
PAΔ3FeΔ*fptX*	*fptX* deletion mutant in PAΔ3Fe	This study
PAΔ3FeΔ*pchHI*	*pchHI* deletion mutant in PAΔ3Fe	This study
PAΔ3FeΔ*fepBCDG*	*fepBCDG* deletion mutant in PAΔ3Fe	This study
PAΔ3FeΔ*fptX*Δ*pchHI*	*fptX*/*pchHI* deletion mutant in PAΔ3Fe	This study
PAΔ3FeΔ*fptX*Δ*pchHI*Δ*fepBCDG*	*fptX*/*pchHI*/*fepBCDG* deletion mutant in PAΔ3Fe	This study
PAΔ3FeΔ*pchHI*Δ*fepBCDG*	*pchHI*/*fepBCDG* deletion mutant in PAΔ3Fe	This study
PAΔ3FeΔ*fptX*Δ*pchHI*Δ*fepC*	*fptX*/*pchHI*/*fepC* deletion mutant in PAΔ3Fe	This study
PAΔ3FeΔ*pqsA*	*pqsA* deletion mutant in PAΔ3Fe	This study
PAΔ3FeΔ*pqsA*Δ*fptX*Δ*pchHI*Δ*fepBCDG*	*pqsA*/*fptX*/*pchHI*/*fepBCDG* deletion mutant in PAΔ3Fe	This study
PAΔ3FeΔ*pqsH*	*pqsH* deletion mutant in PAΔ3Fe	This study
PAΔ3FeΔ*pqsH*Δ*fptX*Δ*pchHI*Δ*fepBCDG*	*pqsH*/*fptX*/*pchHI*/*fepBCDG* deletion mutant in PAΔ3Fe	This study
PAΔ3FeΔ*fptA*Δ*oprF*	*fptA*/*oprF* deletion mutant in PAΔ3Fe	This study
PAΔ3Fe (pME6032)	PAΔ3Fe containing pME6032	This study
PAΔ3FeΔ*fptX*Δ*pchHI*Δ*fepBCDG* (pME6032)	PAΔ3FeΔ*fptX*Δ*pchHI*Δ*fepBCDG* containing pME6032	This study
PAΔ3FeΔ*fptX*Δ*pchHI*Δ*fepBCDG* (pME6032-*fptX*)	PAΔ3FeΔ*fptX*Δ*pchHI*Δ*fepBCDG* containing pME6032-*fptX*	This study
PAΔ3FeΔ*fptX*Δ*pchHI*Δ*fepBCDG*	PAΔ3FeΔ*fptX*Δ*pchHI*Δ*fepBCDG* containing pME6032-*pchHI*	This study

(pME6032-*pchHI*)		
PAΔ3FeΔ*fptX*Δ*pchHI*Δ*fepBCDG* (pME6032-*fepBCDG*)	PAΔ3FeΔ*fptX*Δ*pchHI*Δ*fepBCDG* containing pME6032-*fepBCDG*	This study
PAΔ3FeΔ*fptX*Δ*pchHI*Δ*fepBCDG* (pME6032-*pvdA*)	PAΔ3FeΔ*fptX*Δ*pchHI*Δ*fepBCDG* containing pME6032-*pvdA*	This study
PAΔ3FeΔ*fptX*Δ*pchHI*Δ*fepBCDG* (pME6032-*pchH*I*)	PAΔ3FeΔ*fptX*Δ*pchHI*Δ*fepBCDG* containing pME6032-*pchH*I*	This study
PAΔ3FeΔ*fptX*Δ*pchHI*Δ*fepBCDG* (pME6032-*pchHI**)	PAΔ3FeΔ*fptX*Δ*pchHI*Δ*fepBCDG* containing pME6032-*pchHI**	This study
PAΔ3FeΔ*fptX*Δ*pchHI*Δ*fepC* (pME6032)	PAΔ3FeΔ*fptX*Δ*pchHI*Δ*fepC* containing pME6032	This study
PAΔ3FeΔ*fptX*Δ*pchHI*Δ*fepC* (pME6032-*fepC*)	PAΔ3FeΔ*fptX*Δ*pchHI*Δ*fepC* containing pME6032-*fepC*	This study
PAΔ3FeΔ*fptX*Δ*pchHI*Δ*fepC* (pME6032-*fepC**)	PAΔ3FeΔ*fptX*Δ*pchHI*Δ*fepC* containing pME6032-*fepC**	This study
PAΔ3FeΔ*fptA*Δ*oprF* (pME6032)	PAΔ3FeΔ*fptA*Δ*oprF* containing pME6032	This study
PAΔ3FeΔ*pqsA* (pME6032)	PAΔ3FeΔ*pqsA* containing pME6032	This study
PAΔ3FeΔ*pqsA*Δ*fptX*Δ*pchHI*Δ*fepBCDG* (pBBR1MCS-5)	PAΔ3FeΔ*pqsA*Δ*fptX*Δ*pchHI*Δ*fepBCDG* containing pBBR1MCS-5	This study
PAΔ3FeΔ*pqsH* (pME6032)	PAΔ3FeΔ*pqsH* containing pME6032	This study
PAΔ3FeΔ*pqsH*Δ*fptX*Δ*pchHI*Δ*fepBCDG* (pME6032)	PAΔ3FeΔ*pqsH*Δ*fptX*Δ*pchHI*Δ*fepBCDG* containing pME6032	This study
PAΔ3FeΔ*pqsH*Δ*fptX*Δ*pchHI*Δ*fepBCDG* (pME6032-*fptX*)	PAΔ3FeΔ*pqsH*Δ*fptX*Δ*pchHI*Δ*fepBCDG* containing pME6032-*fptX*	This study
PAΔ3FeΔ*pqsH*Δ*fptX*Δ*pchHI*Δ*fepBCDG* (pME6032-*pchHI*)	PAΔ3FeΔ*pqsH*Δ*fptX*Δ*pchHI*Δ*fepBCDG* containing pME6032-*pchHI*	This study
PAΔ3FeΔ*pqsH*Δ*fptX*Δ*pchHI*Δ*fepBCDG* (pME6032-*fepBCDG*)	PAΔ3FeΔ*pqsH*Δ*fptX*Δ*pchHI*Δ*fepBCDG* containing pME6032-*fepBCDG*	This study
PAΔ3FeΔ*pqsA*-LZ	PAΔ3FeΔ*pqsA* *attB*::P_{lecA}-*lacZ*	This study
PAΔ3FeΔ*pqsA*-PZ	PAΔ3FeΔ*pqsA* *attB*::P_{phzA1}-*lacZ*	This study
PAΔ3FeΔ*pqsA*Δ*fptX*Δ*pchHI*Δ*fepBCDG*-LZ	PAΔ3FeΔ*pqsA*Δ*fptX*Δ*pchHI*Δ*fepBCDG* *attB*::P_{lecA}-*lacZ*	This study
PAΔ3FeΔ*tseF*Δ*fptA*Δ*oprF*-LZ	PAΔ3FeΔ*tseF*Δ*fptA*Δ*oprF* *attB*::P_{lecA}-*lacZ*	This study

PAΔ3FeΔ*tseF*Δ*fptA*Δ*oprF*-PZ	PAΔ3FeΔ*tseF*Δ*fptA*Δ*oprF* *attB*::P_{phzA1}-*lacZ*	This study
PAΔ3Fe-*lacZ*	PAΔ3Fe *attB*::P*tac*-*lacZ*	(1)
PAΔ3Fe-*lacZ* (pME6032)	PAΔ3Fe *attB*::P*tac*-*lacZ* containing pME6032	This study
Δ*pvdA*	*pvdA* deletion mutant in PAO1	(1)
Δ*pvdA*Δ*fptX*Δ*pchHI*Δ*fepBCDG*	*fptX*/*pchHI*/*fepBCDG* deletion mutant in Δ*pvdA*	This study
Δ*pvdA*Δ*pqsH*	*pqsH* deletion mutant in Δ*pvdA*	This study
Δ*pvdA*Δ*pqsH*Δ*pchE*	*pchE* deletion mutant in Δ*pvdA*Δ*pqsH*	This study
Δ*pvdA*Δ*pqsH*Δ*fptX*	*fptX* deletion mutant in Δ*pvdA*Δ*pqsH*	This study
Δ*pvdA*Δ*pqsH*Δ*pchHI*	*pchHI* deletion mutant in Δ*pvdA*Δ*pqsH*	This study
Δ*pvdA*Δ*pqsH*Δ*fepBCDG*	*fepBCDG* deletion mutant in Δ*pvdA*Δ*pqsH*	This study
Δ*pvdA*Δ*pqsH*Δ*fptX*Δ*pchHI*	*fptX*/*pchHI* deletion mutant in Δ*pvdA*Δ*pqsH*	This study
Δ*pvdA*Δ*pqsH*Δ*fptX*Δ*pchHI*Δ*fepBCDG*	*fptX*/*pchHI*/*fepBCDG* deletion mutant in Δ*pvdA*Δ*pqsH*	This study
Δ*pvdA*Δ*pqsH*Δ*pchHI*Δ*fepBCDG*	*pchHI*/*fepBCDG* deletion mutant in Δ*pvdA*Δ*pqsH*	This study
Δ*pvdA*Δ*pqsH*Δ*fptX*Δ*pchH*Δ*fepBCDG*	*fptX*/*pchH*/*fepBCDG* deletion mutant in Δ*pvdA*Δ*pqsH*	This study
Δ*pvdA*Δ*pqsH*Δ*fptX*Δ*pchI*Δ*fepBCDG*	*fptX*/*pchI*/*fepBCDG* deletion mutant in Δ*pvdA*Δ*pqsH*	This study
Δ*pvdA*Δ*pqsH* (pME6032)	Δ*pvdA*Δ*pqsH* containing pME6032	This study
Δ*pvdA*Δ*pqsH*Δ*pchE* (pME6032)	Δ*pvdA*Δ*pqsH*Δ*pchE* containing pME6032	This study
Δ*pvdA*Δ*pqsH*Δ*fptX*Δ*pchHI*Δ*fepBCDG* (pME6032)	Δ*pvdA*Δ*pqsH*Δ*fptX*Δ*pchHI*Δ*fepBCDG* containing pME6032	This study
Δ*pvdA*Δ*pqsH*Δ*fptX*Δ*pchHI*Δ*fepBCDG* (pME6032-*fptX*)	Δ*pvdA*Δ*pqsH*Δ*fptX*Δ*pchHI*Δ*fepBCDG* containing pME6032-*fptX*	This study
Δ*pvdA*Δ*pqsH*Δ*fptX*Δ*pchHI*Δ*fepBCDG* (pME6032-*pchHI*)	Δ*pvdA*Δ*pqsH*Δ*fptX*Δ*pchHI*Δ*fepBCDG* containing pME6032-*pchHI*	This study
Δ*pvdA*Δ*pqsH*Δ*fptX*Δ*pchHI*Δ*fepBCDG* (pME6032-*fepBCDG*)	Δ*pvdA*Δ*pqsH*Δ*fptX*Δ*pchHI*Δ*fepBCDG* containing pME6032-*fepBCDG*	This study
Δ*pvdA*Δ*pqsH*Δ*fptX*Δ*pchHI*Δ*fepBCDG* (pME6032-*pvdA*)	*pvdA*/*pqsH*/*fptX*/*pchHI*/*fepBCDG* containing pME6032-*pvdA*	This study
Δ*pvdA*Δ*pqsH*Δ*fptX*Δ*pchHI*Δ*fepBCDG* (pME6032-*pchH*I*)	Δ*pvdA*Δ*pqsH*Δ*fptX*Δ*pchHI*Δ*fepBCDG* containing pME6032-*pchH*I*	This study

Δ*pvdA*Δ*pqsH*Δ*fptX*Δ*pchHI*Δ*fepBCDG* (pME6032-*pchHI**)	Δ*pvdA*Δ*pqsH*Δ*fptX*Δ*pchHI*Δ*fepBCDG* containing pME6032-*pchHI**	This study
Δ*pvdA*Δ*fptX*Δ*pchHI*Δ*fepBCDG* (pME6032)	Δ*pvdA*Δ*fptX*Δ*pchHI*Δ*fepBCDG* containing pME6032	This study
Δ*pvdA*Δ*fptX*Δ*pchHI*Δ*fepBCDG* (pME6032-*fptX*)	Δ*pvdA*Δ*fptX*Δ*pchHI*Δ*fepBCDG* containing pME6032-*fptX*	This study
Δ*pvdA*Δ*fptX*Δ*pchHI*Δ*fepBCDG* (pME6032-*pchHI*)	Δ*pvdA*Δ*fptX*Δ*pchHI*Δ*fepBCDG* containing pME6032-*pchHI*	This study
Δ*pvdA*Δ*fptX*Δ*pchHI*Δ*fepBCDG* (pME6032-*fepBCDG*)	Δ*pvdA*Δ*fptX*Δ*pchHI*Δ*fepBCDG* containing pME6032-*fepBCDG*	This study
Δ*pvdA*Δ*pqsH-lacZ*	Δ*pvdA*Δ*pqsH attB*::P*tac-lacZ*	This study
Δ*pvdA*Δ*pqsH-lacZ* (pME6032)	Δ*pvdA*Δ*pqsH attB*::P*tac-lacZ* containing pME6032	This study
Δ*pvdA*Δ*pqsH*Δ*pchE*Δ*fptA*	*fptA* deletion mutant in Δ*pvdA*Δ*pqsH*Δ*pchE*	This study
Δ*pvdA*Δ*pqsH*Δ*pchE*Δ*fptX*	*fptX* deletion mutant in Δ*pvdA*Δ*pqsH*Δ*pchE*	This study
Δ*pvdA*Δ*pqsH*Δ*pchE*Δ*fptX*Δ*pchHI*	*fptX*/*pchHI* deletion mutant in Δ*pvdA*Δ*pqsH*Δ*pchE*	This study
Δ*pvdA*Δ*pqsH*Δ*pchE*Δ*fptX*Δ*pchHI*Δ*fepBCDG*	*fptX*/*pchHI*/*fepBCDG* deletion mutant in Δ*pvdA*Δ*pqsH*Δ*pchE*	This study
Δ*pvdA*Δ*pchE*	*pvdA*/*pchE* deletion mutant in PAO1	(1)
Δ*pvdA*Δ*pchE*Δ*fptA*	*fptA* deletion mutant in Δ*pvdA*Δ*pchE*	This study
Δ*pvdA*Δ*pchE*Δ*fptX*	*fptX* deletion mutant in Δ*pvdA*Δ*pchE*	This study
Δ*pvdA*Δ*pchE*Δ*fptX*Δ*fepBCDG*	*fptX*/*fepBCDG* deletion mutant in Δ*pvdA*Δ*pchE*	This study
Δ*pvdA*Δ*pchE*Δ*fptX*Δ*pchHI*	*fptX*/*pchHI* deletion mutant in Δ*pvdA*Δ*pchE*	This study
Δ*pvdA*Δ*pchE*Δ*fptX*Δ*pchHI*Δ*fepBCDG*	*fptX*/*pchHI*/*fepBCDG* deletion mutant in Δ*pvdA*Δ*pchE*	This study
Δ*pvdA*Δ*pchE*Δ*fptX*Δ*pchHI*Δ*fepBCDG*Δ*pchR*	*fptX*/*pchHI*/*fepBCDG*/*pchR* deletion mutant in Δ*pvdA*Δ*pchE*	This study
Δ*pvdA*Δ*pqsH*Δ*pchE* (pME6032)	Δ*pvdA*Δ*pqsH*Δ*pchE* containing pME6032	This study
Δ*pvdA*Δ*pqsH*Δ*pchE*Δ*fptA* (pME6032)	Δ*pvdA*Δ*pqsH*Δ*pchE*Δ*fptA* containing pME6032	This study
Δ*pvdA*Δ*pqsH*Δ*pchE*Δ*fptX*Δ*pchHI*Δ*fepBCDG* (pME6032)	Δ*pvdA*Δ*pqsH*Δ*pchE*Δ*fptX*Δ*pchHI*Δ*fepBCDG* containing pME6032	This study
Δ*pvdA*Δ*pqsH*Δ*pchE*Δ*fptX*Δ*pchHI*Δ*fepBCDG* (pME6032-*fptX*)	Δ*pvdA*Δ*pqsH*Δ*pchE*Δ*fptX*Δ*pchHI*Δ*fepBCDG* containing pME6032	This study
Δ*pvdA*Δ*pqsH*Δ*pchE*Δ*fptX*Δ*pchHI*Δ*fepBCDG* (pME6032-*pchHI*)	Δ*pvdA*Δ*pqsH*Δ*pchE*Δ*fptX*Δ*pchHI*Δ*fepBCDG* containing pME6032-*pchHI*	This study

Δ*pvdA*Δ*pqsH*Δ*pchE*Δ*fptX*Δ*pchHI*Δ*fepBCDG* (pME6032-*fepBCDG*)	Δ*pvdA*Δ*pqsH*Δ*pchE*Δ*fptX*Δ*pchHI*Δ*fepBCDG* containing pME6032-*fepBCDG*	This study
Δ*pvdA*Δ*pqsH*Δ*pchE*Δ*fptX*Δ*pchHI*Δ*fepBCDG* (pME6032-*pvdA*)	Δ*pvdA*Δ*pqsH*Δ*pchE*Δ*fptX*Δ*pchHI*Δ*fepBCDG* containing pME6032-*pvdA*	This study
Δ*pvdA*Δ*pqsH*Δ*pchE*Δ*fptX*Δ*pchHI*Δ*fepBCDG* (pBBR1MCS-5)	Δ*pvdA*Δ*pqsH*Δ*pchE*Δ*fptX*Δ*pchHI*Δ*fepBCDG* containing pBBR1MCS-5	This study
Δ*pvdA*Δ*pchE*-AZ	Δ*pvdA*Δ*pchE* *attB*::P_{fptA}-*lacZ*	This study
Δ*pvdA*Δ*pchE*Δ*fptA*-AZ	Δ*pvdA*Δ*pchE*Δ*fptA* *attB*::P_{fptA}-*lacZ*	This study
Δ*pvdA*Δ*pchE*Δ*fptX*-AZ	Δ*pvdA*Δ*pchE*Δ*fptX* *attB*::P_{fptA}-*lacZ*	This study
Δ*pvdA*Δ*pchE*Δ*fptX*Δ*fepBCDG*-AZ	Δ*pvdA*Δ*pchE*Δ*fptX*Δ*fepBCDG* *attB*::P_{fptA}-*lacZ*	This study
Δ*pvdA*Δ*pchE*Δ*fptX*Δ*pchHI*-AZ	Δ*pvdA*Δ*pchE*Δ*fptX*Δ*pchHI* *attB*::P_{fptA}-*lacZ*	This study
Δ*pvdA*Δ*pchE*Δ*fptX*Δ*pchHI*Δ*fepBCDG*-AZ	Δ*pvdA*Δ*pchE*Δ*fptX*Δ*pchHI*Δ*fepBCDG* *attB*::P_{fptA}-*lacZ*	This study
Δ*pvdA*Δ*pchE*Δ*fptX*Δ*pchHI*Δ*fepBCDG* Δ*pchR*-AZ	Δ*pvdA*Δ*pchE*Δ*fptX*Δ*pchHI*Δ*fepBCDG* Δ*pchR* *attB*::P_{fptA}-*lacZ*	This study
Δ*pvdA*Δ*pchE*-DZ	Δ*pvdA*Δ*pchE* *attB*::P_{pchD}-*lacZ*	This study
Δ*pvdA*Δ*pchE*Δ*fptA*-DZ	Δ*pvdA*Δ*pchE*Δ*fptA* *attB*::P_{pchD}-*lacZ*	This study
Δ*pvdA*Δ*pchE*Δ*fptX*-DZ	Δ*pvdA*Δ*pchE*Δ*fptX* *attB*::P_{pchD}-*lacZ*	This study
Δ*pvdA*Δ*pchE*Δ*fptX*Δ*fepBCDG*-DZ	Δ*pvdA*Δ*pchE*Δ*fptX*Δ*fepBCDG* *attB*::P_{pchD}-*lacZ*	This study
Δ*pvdA*Δ*pchE*Δ*fptX*Δ*pchHI*-DZ	Δ*pvdA*Δ*pchE*Δ*fptX*Δ*pchHI* *attB*::P_{pchD}-*lacZ*	This study
Δ*pvdA*Δ*pchE*Δ*fptX*Δ*pchHI*Δ*fepBCDG*-DZ	Δ*pvdA*Δ*pchE*Δ*fptX*Δ*pchHI*Δ*fepBCDG* *attB*::P_{pchD}-*lacZ*	This study
Δ*pvdA*Δ*pchE*Δ*fptX*Δ*pchHI*Δ*fepBCDG*Δ*pchR*-DZ	Δ*pvdA*Δ*pchE*Δ*fptX*Δ*pchHI*Δ*fepBCDG*Δ*pchR* *attB*::P_{pchD}-*lacZ*	This study
Δ*pvdA*Δ*pchE*-EZ	Δ*pvdA*Δ*pchE* *attB*::P_{pchE}-*lacZ*	This study
Δ*pvdA*Δ*pchE*Δ*fptA*-EZ	Δ*pvdA*Δ*pchE*Δ*fptA* *attB*::P_{pchE}-*lacZ*	This study
Δ*pvdA*Δ*pchE*Δ*fptX*-EZ	Δ*pvdA*Δ*pchE*Δ*fptX* *attB*::P_{pchE}-*lacZ*	This study
Δ*pvdA*Δ*pchE*Δ*fptX*Δ*fepBCDG*-EZ	Δ*pvdA*Δ*pchE*Δ*fptX*Δ*fepBCDG* *attB*::P_{pchE}-*lacZ*	This study
Δ*pvdA*Δ*pchE*Δ*fptX*Δ*pchHI*-EZ	Δ*pvdA*Δ*pchE*Δ*fptX*Δ*pchHI* *attB*::P_{pchE}-*lacZ*	This study
Δ*pvdA*Δ*pchE*Δ*fptX*Δ*pchHI*Δ*fepBCDG*-EZ	Δ*pvdA*Δ*pchE*Δ*fptX*Δ*pchHI*Δ*fepBCDG* *attB*::P_{pchE}-*lacZ*	This study
Δ*pvdA*Δ*pchE*Δ*fptX*Δ*pchHI*Δ*fepBCDG*Δ*pchR*-EZ	Δ*pvdA*Δ*pchE*Δ*fptX*Δ*pchHI*Δ*fepBCDG*Δ*pchR* *attB*::P_{pchE}-*lacZ*	This study
PAO1-AZ	PAO1 *attB*::P_{fptA}-*lacZ*	This study
PAO1Δ*fptA*-AZ	PAO1Δ*fptA* *attB*::P_{fptA}-*lacZ*	This study

PAO1Δ*fptX*-AZ	PAO1Δ*fptX* *attB*::P_{fptA}-*lacZ*	This study
PAO1Δ*fptX*Δ*fepBCDG*-AZ	PAO1Δ*fptX*Δ*fepBCDG* *attB*::P_{fptA}-*lacZ*	This study
PAO1Δ*fptX*Δ*pchHI*-AZ	PAO1Δ*fptX*Δ*pchHI* *attB*::P_{fptA}-*lacZ*	This study
PAO1Δ*fptX*Δ*pchHI*Δ*fepBCDG*-AZ	PAO1Δ*fptX*Δ*pchHI*Δ*fepBCDG* *attB*::P_{fptA}-*lacZ*	This study
PAO1Δ*fptX*Δ*pchHI*Δ*fepBCDG*Δ*pcchR*-AZ	PAO1Δ*fptX*Δ*pchHI*Δ*fepBCDG*Δ*pcchR* *attB*::P_{fptA}-*lacZ*	This study
PAO1-DZ	PAO1 *attB*::P_{pchD}-*lacZ*	This study
PAO1Δ*fptA*-DZ	PAO1Δ*fptA* *attB*::P_{pchD}-*lacZ*	This study
PAO1Δ*fptX*-DZ	PAO1Δ*fptX* *attB*::P_{pchD}-*lacZ*	This study
PAO1Δ*fptX*Δ*fepBCDG*-DZ	PAO1Δ*fptX*Δ*fepBCDG* *attB*::P_{pchD}-*lacZ*	This study
PAO1Δ*fptX*Δ*pchHI*-DZ	PAO1Δ*fptX*Δ*pchHI* *attB*::P_{pchD}-*lacZ*	This study
PAO1Δ*fptX*Δ*pchHI*Δ*fepBCDG*-DZ	PAO1Δ*fptX*Δ*pchHI*Δ*fepBCDG* *attB*::P_{pchD}-*lacZ*	This study
PAO1Δ*fptX*Δ*pchHI*Δ*fepBCDG*Δ*pchR*-DZ	PAO1Δ*fptX*Δ*pchHI*Δ*fepBCDG*Δ*pchR* *attB*::P_{pchD}-*lacZ*	This study
PAO1-EZ	PAO1 *attB*::P_{pchE}-*lacZ*	This study
PAO1Δ*fptA*-EZ	PAO1Δ*fptA* *attB*::P_{pchE}-*lacZ*	This study
PAO1Δ*fptX*-EZ	PAO1Δ*fptX* *attB*::P_{pchE}-*lacZ*	This study
PAO1Δ*fptX*Δ*fepBCDG*-EZ	PAO1Δ*fptX*Δ*fepBCDG* *attB*::P_{pchE}-*lacZ*	This study
PAO1Δ*fptX*Δ*pchHI*-EZ	PAO1Δ*fptX*Δ*pchHI* *attB*::P_{pchE}-*lacZ*	This study
PAO1Δ*fptX*Δ*pchHI*Δ*fepBCDG*-EZ	PAO1Δ*fptX*Δ*pchHI*Δ*fepBCDG* *attB*::P_{pchE}-*lacZ*	This study
PAO1Δ*fptX*Δ*pchHI*Δ*fepBCDG*Δ*pchR*-EZ	PAO1Δ*fptX*Δ*pchHI*Δ*fepBCDG*Δ*pchR* *attB*::P_{pchE}-*lacZ*	This study
Escherichia coli		
TG1	[F´traD36proABlacIqZΔM15]supEthi-1 Δ(lac-proAB) Δ(mcrB-hsdSM)5(rK- mK-)	Laboratory stock
BTH101	*cya*(-) *E. coli*, host for the bacterial two-hybrid system	Laboratory stock
S17-1	RP4-2(Km::Tn7, Tc::Mu-1), pro-82, LAMpir, recA1, endA1, thiE1, hsdR17, creC510	Laboratory stock
**Plasmids**	**Characteristics**	
pK18*mobsacB*	Km^r; *sacB*-based gene replacement vector	(2)
pK18-Δ*fptX*	Km^r, Gm^r; Δ*fptX*::Gm in pK18*mobsacB*	This study
pK18-Δ*pchHI*	Km^r, Gm^r; Δ*pchHI*::Gm in pK18*mobsacB*	This study

pK18-Δ*fepC*	Km^r, Gm^r; Δ *fepC*::Gm in pK18*mobsacB*	This study
pK18-Δ*fepBCDG*	Km^r, Gm^r; Δ*fepBCDG*::Gm in pK18*mobsacB*	This study
pK18-Δ*fptA*	Km^r, Gm^r; Δ*fptA*::Gm in pK18*mobsacB*	(1)
pK18-Δ*pqsA*	Km^r, Gm^r; Δ*pqsA*::Gm in pK18*mobsacB*	(1)
pK18-Δ*pqsH*	Km^r, Gm^r; Δ*pqsH*::Gm in pK18*mobsacB*	(1)
p34s-Gm	Amp^r; Gm resistant cassette carrying vector	(3)
pME6032	Shuttle vector between *Pseudomonas* and *E.coli* containing *lacI*^{q}-P*tac* fragment for gene expression; source of *tetA* gene cassette, Tc^r	(4)
pME6032-*fptX*	*fptX* cloned into pME6032 for complementation	This study
pME6032-*pchH*I*	*pchH*I* cloned into pME6032 for complementation	This study
pME6032-*pchHI**	*pchHI** cloned into pME6032 for complementation	This study
pME6032-*pchHI*	*pchHI* cloned into pME6032 for complementation	This study
pME6032-*fepBCDG*	*fepBCDG* cloned into pME6032 for complementation	This study
pME6032-*fepC*	*fepC* cloned into pME6032 for complementation	This study
pME6032-*fepC**	*fepC**cloned into pME6032 for complementation	This study
pME6032-*pvdA*	*pvdA* cloned into pME6032 for complementation	(1)
pMini-CTX-*lacZ*	Ω-FRT-attP-MCS, ori, int, oriT, Tc^r	(5, 6)
pMini-CTX-P*tac*::*lacZ*	513 bp tac promoter region from pME6032 in pMini-CTX-*lacZ*	(1)
lecA-lacZ	1036 bp upstream region of *lecA* in pMini-CTX-*lacZ*	This study
phzA1-lacZ	1286 bp upstream region of *phzA1* in pMini-CTX-*lacZ*	(7)
fptA-lacZ	705 bp upstream region of *fptA* in pMini-CTX-*lacZ*	This study
pchD-lacZ	684 bp upstream region of *pchD* in pMini-CTX-*lacZ*	(7)
pchE-lacZ	684 bp upstream region of *pchE* in pMini-CTX-*lacZ*	(7)
pKT25	p15A origin of replication encoding $CyaA_{1-224}$; Km^r	Laboratory stock
pUT18C	ColE1 origin of replication encoding $Cya_{A225-399}$; Amp^r	Laboratory stock
pKT25M	Modified pKT25	(8)
pUT18CM	Modified pUT18C	(8)

pKT25-*zip*	Leucine zipper of GCN1 (BTH positive control); Kmr	Laboratory stock
pUT18C-*zip*	Leucine zipper of GCN1 (BTH positive control)	Laboratory stock
pUT18CM-*fptX*	*fptX* in pUT18CM	This study
pUT18CM-*pchH*	*pchH* in pUT18CM	This study
pUT18CM-*pchI*	*pchI* in pUT18CM	This study
pUT18CM-*fepB*	*fepB* in pUT18CM	This study
pUT18CM-*fepC*	*fepC* in pUT18CM	This study
pUT18CM-*fepD*	*fepD* in pUT18CM	This study
pUT18CM-*fepG*	*fepG* in pUT18CM	This study
pUT18CM-*pchHI*	*pchHI* in pUT18CM	This study
pKT25M-*fptX*	*fptX* in pKT25M	This study
pKT25M-*pchH*	*pchH* in pKT25M	This study
pKT25M-*pchI*	*pchI* in pKT25M	This study
pKT25M-*fepB*	*fepB* in pKT25M	This study
pKT25M-*fepC*	*fepC* in pKT25M	This study
pKT25M-*fepD*	*fepD* in pKT25M	This study
pKT25M-*fepG*	*fepG* in pKT25M	This study

Table S2 List of primers used in this study

Name	Sequence (5′→3′)	
fptX Up F	CTCGTCTAGACCAGCGTTTGAGCCTCAG	To generate pK18-Δ*fptX*
fptX Up R	CCAGCATCTCCTGGGACAGGTAGAGCAC	
fptX Low F	CCTGTCCCAGGAGATGCTGGCGTCTTCG	
fptX Low R	CTCGAAGCTTAACTGGGTGTGCATGGTG	
fptX F	CTCGGAATTCATGCTTGAGCTGTACCGCCAC	To generate pME6032-*fptX*
fptX R	CTCGCTCGAGGTGGAACGGCCACGCTAG	
two-hybrid *fptX* F	ATGCAGATCTATGCTTGAGCTGTACCGCCAC	To generate pUT18CM-*fptX* or pKT25M-*fptX*
two-hybrid *fptX* R	TCGACTCGAGAAGATCATCCTGGCCAAC	
pchHI Up F	CTCGGGATCCTGCCTGCTCAACACCTTC	To generate pK18-Δ*pchHI*
pchHI Up R	GTACCTGGTCGGGCAACAGCGAGCAGAG	
pchHI Low F	GCTGTTGCCCGACCAGGTACTGCTGCTGG	
pchHI Low R	CTCGAAGCTTTTGCGCACCAGGTTGACG	
SD-*pchHI* F	CTCGGGTACCCCCTGTGGCAGCGGATCG	To generate pME6032-SD-*pchHI*
SD-*pchHI* R	CTCGAGATCTCGATTGCCGGTCTTTCTC	
SD-*pchH*I* Up F	CTCGGGTACCCCCTGTGGCAGCGGATCG	To generate pME6032-SD-*pchH*I*
SD-*pchH*I* Up R	TCGAGCAGCAACAGGCTCGCCGGCGATAG	
SD-*pchH*I* Low F	GAGCCTGTTGCTGCTCGACGCCCCAACCTCGGCGCTGGATC	
SD-*pchH*I* Low R	CTCGAGATCTCGATTGCCGGTCTTTCTC	
SD-*pchHI** Up F	CTCGGGTACCCCCTGTGGCAGCGGATCG	To generate pME6032-SD-*pchHI**
SD-*pchHI** Up R	GGTGGGCGCGTCGAGCAGCAGCAACGG	
SD-*pchHI** Low F	CTGCTCGACGCGCCCACCGCCAGCCTCGAC	
SD-*pchHI** Low R	CTCGAGATCTCGATTGCCGGTCTTTCTC	
three -hybrid *pchHI* F	ATGCAGATCTGTGACCCCGGTGCTGTGG	To generate pUT18CM-*pchHI*
three -hybrid *pchHI* R	GTCATCTAGACGATTGCCGGTCTTTCTC	
two-hybrid *PchH* F	ATGCAGATCTGTGACCCCGGTGCTGTGG	To generate pUT18CM-*pchH* or pKT25M-*pchH*
two-hybrid *pchH* R	TCGACTCGAGCCAGCAATACGCCGCAAG	
two-hybrid *PchI* F	GTCAGGATCCATGACCCTGTTCGAACGAATG	To generate pUT18CM-*pchI*

two-hybrid *pchI* R	GCTAGTCGACTATACGATTTGATAATGC	or pKT25M-*pchI*
fepBCDG Up F	CTCGGGATCCTCACACAGGGGAATCGTC	To generate pK18-Δ*fepBCDG*
fepBCDG Up R	CCACCAGACGCTGCTCTGGCTACTGCTG	
fepBCDG Low F	CCAGAGCAGCGTCTGGTGGAGGAGGTG	
fepBCDG Low R	CTCGAAGCTTCTGCAGAGCCTGGTGAAG	
fepBCDG F	CTCGGAATTCCAGCCTGGGCGGTCGTTG	To generate
fepBCDG R	CTCGAGATCTGCCGCGCTGAGCCACCTC	pME6032-*fepBCDG*
fepC Up F	ATCGAGATCTGTCGAAGCGCTGGATGGC	To generate pK18-Δ*fepC*
fepC Up R	GTGAAGATATGGGTCGACTTGCCGCAGG	
fepC Low F	AAGTCGACCCATATCTTCACCGAGCGTC	
fepC Low R	ATCGAAGCTTAGAGCCTGGTGAAGGAAG	
fepC F	AGCTGAATTCATGACCCATCGCCTGCAC	To generate pME6032-*fepC*
fepC R	ATGCAGATCTGTACTCCACCCCGGCATG	
*fepC** Up F	AGCTGAATTCATGACCCATCGCCTGCAC	To generate pME6032-*FepC**
*fepC** Up R	TGGTTGGCGCGTCGAGCAGCAGCAACGG	
*fepC** Low F	TGCTCGACGCGCCAACCACCTACCTGGATATCGTCCAC	
*fepC** Low R	ATGCAGATCTGTACTCCACCCCGGCATG	
two-hybrid *fepB* F	ATGCAGATCTATGCCGACCCGCCGCCGCTCC	To generate pUT18CM-*fepB*
two-hybrid *fepB* R	TCGACTCGAGCAGGGCATCCAGGGTGAC	or pKT25M-*fepB*
two-hybrid *fepC* F	GTCAGGATCCATGACCCATCGCCTGCAC	To generate pUT18CM-*fepC*
two-hybrid *fepC* R	TCGACTCGAGGCAGGTCAACCAGTCCAC	or pKT25M-*fepC*
two-hybrid *fepD* F	ATGCAGATCTATGCAAGCGTCCCCGATG	To generate pUT18CM-*fepD*
two-hybrid *fepD* R	TCGACTCGAGACGCAGCGACAGGGAACC	or pKT25M-*fepD*
two-hybrid *fepG* F	GTCAGGATCCATGAACGGTCTCCACGCCCTG	To generate pUT18CM-*fepG*
two-hybrid *fepG* R	TCGACTCGAGTCTCGACCATCCCCATGC	or pKT25M-*fepG*
lecA F	CTCGCTCGAGTGTGTTTCCTGGCGTTCAG	To generate *lecA*-*lacZ*
lecA R	CTCGCTGCAGCTGGGTAGGTCCGTAACTG	
fptA F	TGACGGTACCGCGTTGCCTGGAACCTGC	To generate *fptA*-*lacZ*
fptA R	AGCTAAGCTTCACCATGTCCGGCAGTTC	

Table S3 Identification of FptX interacting proteins

FptX binding proteins selected by bacterial two-hybrid

Gene No.	Description	Hit times[a]
PA4159	Iron-enterobactin transporter periplasmic binding protein FepB	10
PA4160	Ferric enterobactin transporter FepD	9
PA4161	Ferric enterobactin transporter FepG	7
PA3299	Long-chain fatty acid coenzyme-A ligase FadD1	6

[a] Number of times each protein was identified in the two-hybrid screen.

1. Lin J, Zhang W, Cheng J, Yang X, Zhu K, Wang Y, Wei G, Qian PY, Luo ZQ, Shen X. 2017. A *Pseudomonas* T6SS effector recruits PQS-containing outer membrane vesicles for iron acquisition. Nature Communication 8:14888.
2. Schafer A, Tauch A, Jager W, Kalinowski J, Thierbach G, Puhler A. 1994. Small mobilizable multi-purpose cloning vectors derived from the *Escherichia coli* plasmids pK18 and pK19: selection of defined deletions in the chromosome of Corynebacterium glutamicum. Gene 145:69-73.
3. Dennis JJ, Zylstra GJ. 1998. Plasposons: modular self-cloning minitransposon derivatives for rapid genetic analysis of gram-negative bacterial genomes. Appl Environ Microbiol 64:2710-5.
4. Heeb S, Blumer C, Haas D. 2002. Regulatory RNA as mediator in GacA/RsmA-dependent global control of exoproduct formation in *Pseudomonas fluorescens* CHA0. J Bacteriol 184:1046-56.
5. Becher A, Schweizer HP. 2000. Integration-proficient *Pseudomonas aeruginosa* vectors for isolation of single-copy chromosomal *lacZ* and *lux* gene fusions. Biotechniques 29:948-50, 952.
6. Hoang TT, Kutchma AJ, Becher A, Schweizer HP. 2000. Integration-proficient plasmids for *Pseudomonas aeruginosa*: site-specific integration and use for engineering of reporter and expression strains. Plasmid 43:59-72.
7. Lin J, Cheng J, Chen K, Guo C, Zhang W, Yang X, Ding W, Ma L, Wang Y, Shen X. 2015. The *icmF3* locus is involved in multiple adaptation- and virulence-related characteristics in *Pseudomonas aeruginosa* PAO1. Frontiers in Cellular and Infection Microbiology 5:70.
8. Xu S, Peng Z, Cui B, Wang T, Song Y, Zhang L, Wei G, Wang Y, Shen X. 2014. FliS modulates FlgM activity by acting as a non-canonical chaperone to control late flagellar gene expression, motility and biofilm formation in *Yersinia pseudotuberculosis*. Environ Microbiol 16:1090-104.
